# The Use of the Exploratory Sequential Approach in Mixed-Method Research: A Case of Contextual Top Leadership Interventions in Construction H&S

**DOI:** 10.3390/ijerph19127276

**Published:** 2022-06-14

**Authors:** Siphiwe Gogo, Innocent Musonda

**Affiliations:** 1 Postgraduate School of Engineering Management, University of Johannesburg, Cnr Kingsway & University Roads, Auckland Park, Johannesburg 2092, South Africa; 2 Department of Construction Management and Quantity Surveying, University of Johannesburg, Cnr Kingsway & University Roads, Auckland Park, Johannesburg 2092, South Africa; imusonda@uj.ac.za

**Keywords:** exploratory sequential, mixed-method, qualitative data, quantitative data, reliability and validity, top safety leadership, safety leadership in construction, transformation of safety culture, safety culture in construction, top leadership

## Abstract

Quality and rigour remain central to the methodological process in research. The use of qualitative and quantitative methods in a single study was justified here against using a single method; the empirical output from the literature review should direct the current worldview and, subsequently, the methodologies applied in research. It is critical to gather contextual behavioural data from subject matter experts—this helps establish context and confirm the hypotheses arising from the literature, which leads to the refinement of the theory’s applicability for developing a conceptual model. This paper identified the top leaders in construction organisations as subject matter experts. Nine semi-structured interviews were conducted, representing the South African construction industry grading. The output of the refined hypothesis was followed by a survey that targeted *n* = 182 multi-level senior leaders to gather further perspectives and validate the conceptual model. The outcome resulting from the rigorous validation process adopted—the analysis process, which included Spearman rank correlation, ordinal logistic regression and multinomial generalised linear modelling—demonstrated that the lack of H&S commitment in top leadership persists, despite high awareness of the cruciality of H&S in their organisations. Contextual competence, exaggerated by the local setting, is one source of this deficiency. This paper provides guidelines for using the exploratory sequential approach in mixed-method research to effectively deal with contextual issues based on non-parametric modelling data in top leadership H&S interventions.

## 1. Introduction

Edmonds and Kennedy [1] defined the exploratory sequential technique as a progressive strategy that is used anytime that quantitative (QUAN) results are augmented by qualitative (QUAL) data. As a result, quantitative data analyses and explains the QUAL results in succession. The exploratory sequential technique is distinct from the explanatory sequential technique because it explores a concept before validating it, allowing for greater versatility in discovering novel ideas offered by the QUAL approach [2]. Numerous projects characterised by novel instrument creation choose this method as it enables the scholar to construct the instrument using QUAL information and afterwards verify it quantitatively [1,3]. As the sort of information generated by the first phase is uncertain—including whether it will emerge in a deterministic or non-parametric framework—and because the first phase is undertaken on a limited sample size, even though saturation would be achieved, the development of a new measurement instrument will be required. This is undertaken to handle the complexities of the resultant model characteristics because the contextual setting of top leadership is uncertain of the H&S culture’s consequences. Categorical data enables a greater level of precision and unambiguity [4]. Hence, it is advisable to perform validation or tests on the QUAN part of the model [3,5].

One of the distinct advantages of using an exploratory sequential approach is described by Heesen et al. [6] as a method that comparatively provides more robust validity. First, according to Flick, the interview-based QUAL methodological technique is suitable for resolving unresolved issues and developing and extending ideas based on such discoveries [7]. Interviews generate extensive data that allows subdomains of ideas to be studied. Furthermore, interviews are a direct data-collecting approach that is optimum for understanding issues’ complexity and depth. These collected ideas stemming from the rich data collected are used to reinforce the hypothesis [8]. When referring to the survey QUAN methodological approach, Bajpai [9] asserts that primary sources of data provide multitudes of benefits; it is noted that primary findings are frequently pertinent to the research objectives since they are collected on an individual basis. Applying both QUAL and QUAL approaches to single research works offers a more significant opportunity to establish more insight into the study subject, whilst a higher degree of validity and accuracy are achieved compared to applying a single approach [10,11].

This paper presents an interpretative, exploratory sequential methodology established on contextualism/a pragmatic worldview. Therefore, it is critical to establish the basis for this worldview as a start, to create a platform for the type of knowledge approach that this paper has adopted.

## 2. Theory

### 2.1. Establishing the Worldview

According to Crotty [12], a worldview or ontology is how the world is interpreted as existing. Research indicates the difficulty caused by the environmental context in the infrastructural development initiative in South Africa—particularly the necessary competency in upper-echelon leaders to lead a high-performance culture in organisational H&S [13]. In research associated with this, failure to select an appropriate tool in the beginning further increases methodological difficulties and causes severe confusion, leading to worthless study outputs [14,15]. Dumrak et al. [16] and Marle and Vidal [17] emphasise the complications brought by context by pointing to the extreme intricacies of major construction projects compared to smaller projects.

Accordingly, in a literature review, this study has applied an approach promoting contextualisation, according to Pepper [18]. This theoretical paradigm is therefore applied systematically throughout the whole study. Perception may be classified into four theoretical aspects: formism, mechanism, organicism, and contextualism, according to the orientation to cognition by Pepper [18]. Contextualism is a theoretical paradigm that presupposes a definitive understanding of a phenomenon categorisation occurs once it is placed in its main context [18]. As a result, it is logical for this paper to be aligned to a worldview that demonstrates the effects of the national and economic sectoral environment on the capacity of top leadership to change H&S culture and influence H&S results.

Zikmund [19] views contextualism as pragmatism, asserting that pragmatism as a philosophy is based on behaviour, circumstances, and outcomes rather than past conditions. It is supported by a paradigm focused on what constitutes logic and how to resolve issues.

### 2.2. Epistemology

True perception is seldom universal but rather illative, interpretative, and speculative. The criterion by which existence is measured is mostly pragmatic [3,14,20]. Crotty [12] describes epistemology as a conceptual viewpoint followed by a logical position that informs methodology and thus brings purpose to a technique that specifies the study’s logic and variables selection. The respondent (knower) and the individual cognitive bias (the known) in the H&S leadership commitment, in the context defined by the worldview, are the criteria in this case. As Morris [21] suggests, this “known” knowledge acts as a precursor to the efficacy of the interpretative paradigm, which is based on the notion that all knowledge is contextual. The interpretative method is concerned with perceiving nature via one’s subjective impressions. These rely on a mutual engagement between the researcher and the issues and use perception procedures (rather than QUAN) such as interviews. This approach backs up the notion that substance is formed via first-hand opinion; it believes that forecasts are difficult to come by. Per this concept, individuals have free will, aspirations, emotions, and thinking [11,22]. By conducting interviews with subject matter experts—the top leaders—and by also conducting surveys with the top management team (TMT), this study fits well within the interpretative scientific logic concerning the nature of knowledge—hence the adoption of the exploratory sequential technique for gathering and analysing the required data.

### 2.3. Research Design

**Figure 1:**
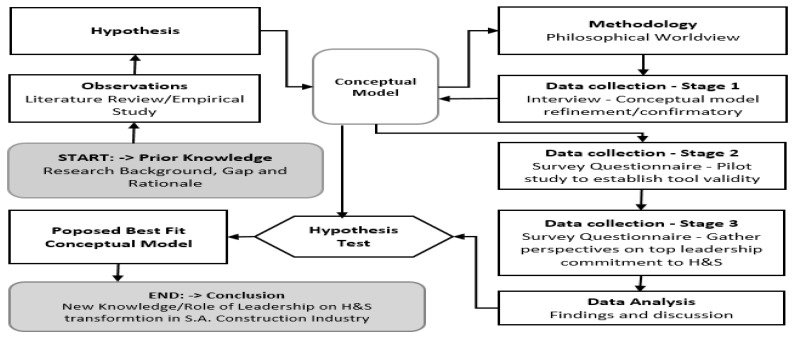
Research design outline (Adapted from Zikmund et al. [19]).

Research design is a thorough description of the steps that must be followed during the data gathering and analysis to produce a satisfactory answer to research questions [5,23]. Additionally, research design may be defined as the overarching principle that the study will adhere to for the many components of the study to be applied logically and succinctly, assisting the scholar in reaching an ideal outcome [24]. Figure 1 shows the research design for this paper.

To accomplish a best-fit conceptual model, which is one of the outputs of this paper, the procedure for adopting the conceptual model’s hypotheses, description, and assessment requirements follow Elangovan and Rajendran’s [25] seven-step rigorous conceptual modelling framework and integrate an acclimation of Zikmund et al.’s [19] scientific approach. The background knowledge was derived from the literature review’s output and synthesised into eight hypotheses, forming a typology. Gogo and Musonda [26] state that this typology forms the basis for the background input for the methodology section described in this paper. The literature demonstrated that leadership is implicitly and explicitly related to H&S outcomes. The eight hypotheses developed from the literature review showed that leadership positions are challenging and require greater comprehension to ensure a decrease in the rates of injury to workers. These hypotheses are all anchored on the actions emanating from top leadership commitment in the South African construction industry.

### 2.4. Conceptual Typology

The functional content measure for the typology in Table 1 is the synthesis of the project’s eight propositions and how these contribute to one another and the study’s four core principles, namely: top leadership participation, regional cultural background, H&S culture and H&S performance. The H&S competency creation and audit framework contribute to top leadership engagement in the typology. In contrast, external influences such as regional culture and the business field are used as the backdrop for top leadership engagement. Table 1 shows the interactions between the model variables.

The contextualism approach to philosophy is reinforced by the national culture and construction industry for this typology. The description of conceptual restrictions by Babbie [27], which range between Meta (South Africa), Macro (Construction sector), Meso (Top organisational leadership) and Micro (Leadership commitment), is applied consistently in the typology [26].

## 3. Methods

### 3.1. Data Collection Approaches

The initial phase of the data collection and analysis was the interview stage. It was characterised by non-probability purposive sampling to establish a theory based on the conceptual model and hypothesis. This was the QUAL phase, where the nine interviews were conducted. The second phase, QUAN, comprised the pilot stage of the survey study, where non-probability convenience sampling was applied to 10% of the target sample as a pilot study to establish the tool’s validity. The third stage, characterised by random probability sampling, used the developed survey questionnaire to gather perspectives on top leadership commitment to H&S by applying QUAN.

### 3.2. Population and Sampling of the QUAL Study

Patten and Newhart [28] describe a population in research as the people, things, wildlife and vegetation involved in the research. Typically, a sample is drawn to represent the population [29]. This paper considered the target population as the top leaders of the construction organisations in South Africa at all nine levels of the CIDB.

Representation, subject-matter expertise and thematic saturation are critical for determining sample sizes [30]. For qualitative studies, Guest et al. [31] describe the adequacy of a sample size as reaching saturation at 6 to 12—where 30 has been defined as the upper limit. To reach QUAL saturation in this paper, a sample of *n* = 9 was aimed for. Additionally, in the QUAL study, subject-matter expertise was ensured by targeting top organisational leaders for their primary prowess in the upper-echelon leadership of contractors and, in particular, their H&S business liability, as per Galvin [32]. Although the target sample for reaching QUAL saturation is small, it still has to fulfil the representation criteria to ensure that the population is well represented [29,32]. In the case of the QUAL study, this representation is achieved by first ensuring that all nine levels of the CIDB are included, and then secondly, by applying a non-probability, purposive sampling method. Non-probability purposive sampling creates a direct method for targeting a subject-matter expert based on defined criteria (CIDB grade, position in company, legal appointment in company and more) [31].

### 3.3. Population and Sampling of the QUAN Study

Consistency is key to quantitative studies [33]. Accordingly, comprehensive insight into a specific phenomenon is validated by many respondents, demonstrating consistency in supporting a defined proposition. Cooper and Schindler [2] posit that of the many variables that define a sample size, the size of the population, uncertainty, variance and confidence interval are among the most influential.

In the QUAN study of this paper, a pilot survey that targeted *n* = 18 respondents was achieved by non-probability convenience sampling. The main survey applied random probability sampling to *n* = 180 respondents. Non-probability convenience sampling was selected for its versatility and to limit the selection of multiple members in the same group—thus ensuring full representation in a smaller sample size. On the other hand, random probability sampling was selected because the likelihood of each member being selected is known—thus ensuring greater participation in the larger sample size [22,33,34]. This is particularly useful when targeting multi-level respondents, as was the case in this paper, to ensure that perspectives in top leadership commitment to H&S are gathered from all levels of the upper-echelon construction organisations’ leadership.

### 3.4. Interview Data Collection Procedure

To ensure clarity in the keynote and the spheres of the enquiry and assessment, Arksey and Knight [35] support the idea of two interviewers; however, this view is refuted by Whiting [36], who posits that the use of a single interview conductor is sufficient. Whiting [36] substantiates this assertion by further providing robust interview guidelines. This paper uses a single interviewer following Whiting [36].

For this study, the design of the semi-structured interview questionnaire followed the protocols and guidelines by Scheele and Groeben [37], Graneheim and Lundman [38] and Whiting [36], which emphasise that questions should be based on the reviewed literature. Accordingly, all interview questions are based on a conceptual typology proposed by Gogo and Musonda [26], which depicts how each leadership aspect relates to each respondent’s H&S aptitude for each contextual determinant. This allows the researcher to examine how the model depicts leadership commitment related to H&S in its full context by accurately representing the respondent [39].

Furthermore, these guidelines included the setting, which in the case of this paper was the respondent’s office—or online in case of constraints for a physical meeting. The respondents were also provided with a short description of the research, and the purpose of the interview was explained clearly. The divisions of the interview questionnaire were explained, and the entire meeting session was kept aligned with the ethical boundaries set beforehand. The interviews were verbal while incorporating the probing techniques shown in Table 2 to reach a clear response. The respondent’s answers were recorded verbatim on both tape and interview answer sheets by the interviewer. Recording the interview answers verbatim offers a robust method for data collection [3].

### 3.5. Survey Data Collection Procedure

Bajpai [9] posits that a comprehensive review consists of primary and secondary data. The secondary data collected is an input for the survey method for the primary data collection. It is important to mention that while primary data is typically gathered on a case-by-case basis, it generally is closely tied to the research aims and questions [9]. According to Cooper and Schindler [2], there are various methods for collecting primary data, but surveys are the most robust method for quantitative data collection. Employing primary data for analysis has numerous advantages, but it also has certain limitations. First, it requires a lot of time, funds, and human resources; however, getting data in some contexts may be problematic due to privacy and security considerations that hinder people from engaging in data collection endeavours. This situation is often overcome by using anonymous surveys [2].

According to Bajpai [9], the tool used to gather data must be dependable and repetitive to be useful. In addition, researchers argue that this tool must fulfil stringent validity criteria, such as reliability and responsiveness, to be regarded as a robust measuring device. During the sample period, survey questionnaires were the primary means of collecting data. Participants in the study were asked to complete a survey in which the test variables and research topics were addressed [40]. As a result of the survey, a quantifiable framework for assessing senior leadership commitment to H&S in construction work was established to help with future research.

For this paper, Google Forms^TM^ was selected as the survey tool because while it is offered for free, it comes with a user-friendly interface for both the respondent and the researcher. It also comes with a myriad of tools, such as graphs, and it can output the captured data to an M.S. Excel spreadsheet for further processing. This tool also offers better validity for collected data than paper surveys because it automatically prevents the respondent from making invalid selections. The researcher produced a simple, short set of guidelines to precede every survey section to ensure understanding of the context and answering requirements [41].

The request for survey participation was administered via email to *n* = 18 (ideally, two for each CIDB level) respondents for the pilot study and *n* = 180 (ideally, 20 to represent each CIDB level) respondents for the actual multi-level perspective study. The same target group of top leaders in the construction industry were targeted for participation. In the request, a link to the online survey was provided.

### 3.6. Ethical Considerations Regarding Data Collection

To begin with, it is necessary to discuss the study’s ethical implications [42]. According to Hay [42], the ethical concepts of justice, beneficence, non-malfeasance, and respect must be included in any investigation conducted. This ensures that protection measures for participants and the institution are addressed. For this paper, this is particularly amplified by the ethical requirements of the University’s policies. These ethical considerations were discussed thus:

- **Ethical intent to achieve autonomy**—brief instructions were provided in the interview and survey questionnaire forms to ensure that the respondents were as autonomous as possible and that dependence on the interviewer was limited.
- **Ethical intent to achieve beneficence**—beneficence is how the study will benefit. For this paper, this was demonstrated by the novelty of the mixed method presented and how this method led to the fulfilment of the research objectives.
- **Ethical intent to achieve non-maleficence**—To ensure just and unbiased participation, demographical information about gender, race, political affiliation, religious beliefs, ethnicity, family orientation, marital status and health conditions of each respondent was not considered or collected. Additionally, ranges of experience rather than discreet numbers were used to provide uniformity among the respondents.
- **Ethical intent to achieve justice**—The risks for participants were covered by a disclaimer and the voluntary participation of the participants, as well as their anonymity. All human rights defined by state laws to institutional laws were observed. The selection process applied for the respondents ensured that the participation of top organisational leaders was inclusive of all groups, without consideration of any form of segregation or target (blind process).

### 3.7. Validity of the Collected Data

For validity, an instrument must be able to accurately compute the value it is meant to ascertain [43]. The internal consistency of collected data is also critical for its validity [22]. Furthermore, the collected data must fulfil the minimum requirements defined by the sampling method in terms of quantity and form [44]; this is particularly useful in dealing with erroneous or missing data. In this study, validity was approached by adopting the analysis of variance, where at least 90% has been set as the cut-off point for valid responses in both the interview and the survey data collection phases. Similar to grounded theory, the thoroughness of the procedure adopted determines the validity of the findings [7,45,46,47].

The online data collection tool adopted for the survey questionnaires prevented the participants from improper selections and ensured that only valid options were selectable from the Likert scale survey questions. This ensured that all submitted forms contained upwards of 95% acceptable data. Furthermore, using a 5-point Likert scale for measurements ensured that the extremities of the data were catered for, whilst a middle ground was also provided for respondents that had a somewhat equal distribution between the extremities in certain questions.

The interview answers’ verbatim transcription, completeness, language, and relevance are also critical for validity criteria [41]. This means that the selection of recording media becomes critical at this stage. For this study, validity was achieved using tape recording and interview answer sheets, which the interviewer consistently completed in all nine interviews with the top leaders. Furthermore, using the probing techniques described in Table 3 ensured that the respondents provided complete and relevant responses to each question.

### 3.8. Reliability of the Collected QUAL Data

Academic assessment systems must provide reliable and accurate data to ensure repeated performance verification [48]. A study’s perceived reliability is bolstered by the precision with which its data were collected and coded (McHugh, 2012). This study adopted a coding process by Adu [49]; however, for reliability, it followed the seven-stage Framework Method by Gale et al. [50], as shown in Table 3. It follows that the intercoder reliability method described and recommended by Freelon [51], Neuendorf [52], Mayring [53], Krippendorff [44] and Hayashi et al. [48], amongst others, was adopted in this study. Everitt and Skrondal [54] and Krippendorff [55] describe the inter-rater agreement as to the coordination level between multiple investigators, assessors, or empirical evaluations. This approach was selected to ensure that sources of errors in coded interview data were eliminated or minimised.

**Table 3:** Intercoder framework method.

Stage	Description	Specifics for This Study
1	Transcription of interview data	The process used to record the interview data during the interviewing phase is interview questionnaires (response spaces).
2	Familiarisation with the interview transcripts	In this case, understanding the transcripts and typing the information into M.S. Excel for each transcript.
3	Coding of the interview data	In this case, the coding process followed the process defined by Adu (2019) and is thoroughly described.
4	Development of a framework for analysis	Intercoder reliability steps as described in the methods and processes, which follow Marying (2014) and Adu (2019).
5	Application of the framework of the analysis	In this case, an understanding of the tool and its application was developed and applied. The tool of choice was Atlas.ti^®^.
6	Data insertion into clusters in the framework	The process for preparing the data for import into Atlas.ti^®^ and then starting the process of coding within this framework.
7	Interpretation of the interview data	The final output, inclusive of the finalisation of the intercoder, revisits and inclusion of inductive codes that emerged throughout the process.

Source: Adopted from Gale et al. [50].

The sophistication of the coding procedure affects the likelihood of mistakes in the data-coding stage [56]. Non-exclusive coding methods are more subject to problems. Although Adu [49] has suggested that a single method for intercoder reliability would suffice, in this paper, several methods were used, following the suggestions from Freelon [51] for providing a strong estimate of reliability.

To achieve the intended multiplatform intercoder reliability, a web-based intercoder reliability calculation platform, ReCal™—developed by Freelon [51]—was selected and then applied for calculating the intercoder reliability. A multi-tool intercoder reliability approach applied Percentage Agreement, Scott’s Pi coefficient, Cohen’s Kappa coefficient and Krippendorff’s Alpha coefficient accordingly in this study.

**(a)**  **Percent agreement**

Percent agreement is defined by Hayes and Krippendorff [41] as a framework for assessing reliability in which two raters select the proportion of elements with comparable attributes. Using this metric, two raters may be distinctive in the form of a percentage [57]. The following formula gives the Percentage Agreement:

PAo = A/n(1)

where: PAo = Observed magnitude of agreement; A = Number of unanimities between the coders; and n = Total number of decisions between the coders.

In this reliability measure, the principle recommended by numerous scholars suggests that the ranges of 75% to 90% are permissible in terms of the proportion of arbitrary consensus [58]. This is the first measure of reliability applied to the QUAN data in this paper. However, this metric does not give a strong level of confidence for reliability precision since it is straightforward and excludes chance as a consideration [41,59]. Yet, its utility remains intact, and as a result, it is appropriate to utilise and include it in this assessment [52].

**(b)**  **Holsti’s Method**

While Holsti’s Method is a variation of the Percentage Agreement, Wang [60] states that if both coders use the same coding units, the findings of this approach will be identical to that of the Percentage Agreement. It would also use the same formula as that of the Percentage Agreement; however, should the coders code different datasets, the following formula is applicable:

PAo = 2A/(N1 + N2)(2)

where: PAo = Observed magnitude of agreement; A = Number of unanimities between the coders; and N1/N2 = Total number of decisions for each respective coder.

This research was designed so that the same set of data was coded individually by the two coders; hence the deployment of Holsti’s Method was not evaluated and was predicated on Percentage Agreement, as indicated by Wang [60]. The Percentage Agreement that was utilised is therefore sufficient.

**(c)**  **Scott’s Pi (π)**

Krippendorff [44] presents Scott’s Pi as an enhancement of the fundamental Percentage Agreement that addresses the predicted consensus amongst the coders for objects that are not tied quantitatively to their descriptions. Percentage Agreement and Holsti’s Method lack the consensus of probability that this metric, which considers the weight of the collective viewpoints, gives [60]. Reliability rigour is seen as having a crucial role in chance [41,52,53]. Landis and Koch [61] used comparative intensities in the attained coefficient to show the gauge of acceptance in reliability while utilising Scott’s Pi. Even though the technique supplied by these authors is optional, it provides good guidance and a benchmark for assessing the robustness of intercoder efficiency when employing both Scott’s Pi and Cohen’s Kappa. Table 4 shows the approach by Landis and Koch [61] in the acceptance criteria of the achieved coefficients.

In this paper, Scott’s Pi is applied without considering the confidence interval; however, a confidence interval is supposed to demonstrate how high the achieved reliability can get.

**(d)**  **Cohen’s Kappa (κ)**

Everitt and Skrondal [54] explain Cohen’s Kappa as a matrix eventuality tabular array that determines the percentage probability of data points, bringing consensus by taking likelihood into account. Interrater reliability testing relies heavily on this powerful statistical tool [57]. Like Scott’s Pi, Cohen’s Kappa has an unweighted formula (without a confidence interval). There are several ways to solve the issue of a rating between more than two raters, including Fleiss kappa; however, for this paper, Cohen’s Kappa will suffice [57]. There is an important distinction between the two: unlike Cohen’s Kappa, Fleiss kappa does not have enforced weighting [52]. Using confidence intervals, a statistician may begin to evaluate the utility of the obtained Kappa, according to McHugh [57]. To show rigour in kappa values, confidence intervals must be utilised instead of Percentage Agreement, which is an exact indication and not an estimate. Confidence Intervals (C.I.s) are described by Sim and Wright [62] and Mukherjee et al. [63] as the degree of trust, which entails that the CI has to be specified before the review of the results. For social research, a lower limit (CI_LL_) and upper limit (CI_UL_) CI of 95% is ordinarily used [63]. Confidence intervals use this formula:

(3) $$CI=\overset{-}{x}\pm z\frac{s}{\sqrt{n}}$$

where: *CI* = Confidence interval (coefficient); X = Sample mean; *z* = Confidence level value; *s* = Sample standard deviation; and *n* = Sample size.

**(e)**  **Krippendorff’s Alpha (α)**

By drawing or assigning probabilistic variables amongst ordinary, unstructured elements, Krippendorff [55] defines Krippendorff’s alpha (*α*) as an internal consistency coefficient that measures the consensus of raters or research instruments to demonstrate validity. In content analysis, Krippendorff’s alpha (*α*) is widely considered among the more precise and adaptable agreement metrics, which provides substantial dependability and rigour (Krippendorff, 2018). Compared to other specialised coefficients, Krippendorff’s alpha offers a more general technique. This allows for a wide range of measurements typically neglected by conventional assessments, such as contrasts between multiple raters, discarding missing data, adjusting to varied test ranges (nominal, ordinal, ratio and interval) and allowing for comparisons across an extensive range of measures [41,48]. The formular for Krippendorff’s Alpha (α) is given by:

α = 1 − (PAo/PA_E_) (4)

where: *α* = Magnitude of agreement (coefficient);

PAo = Observed magnitude of disagreement for analysis values. PAo is given thus:

(5) $$\mathrm{PAo}=\frac{1}{n}\underset{c}{\sum }\;\underset{k}{\sum }\;o_{ck\;\mathrm{metric}\;}\delta_{ck}^{2}$$

PA_E_ = magnitude of disagreement anticipated through chance, given thus:

(6) $${\mathrm{PA}}_{E}=\frac{1}{n\left(n-1\right)}\underset{c}{\sum }\;\underset{k}{\sum }\;n_{c}\cdot n_{k\;\mathrm{metric}\;}\delta_{ck}^{2}$$

Similar to Cohen’s Kappa (κ), for Krippendorff’s alpha (α) values, a confidence interval (CI) of 95% was introduced in this paper. This choice of CI shows that Krippendorff’s alpha reliability indicator is both reliable and dependable [44,53]. For the acceptance of the test results, the number of values was 0 to 1, with 0 representing absolute conflict and 1 representing absolute consensus. Krippendorff [44] posits that it is typical to expect an alpha value of 0.800 as an acceptable baseline, while 0.667 can be regarded as the lower reasonable threshold (L.L.) for which preliminary assumptions are permissible.

### 3.9. Reliability of the Collected QUAN Data

The term “reliability” refers to the consistency of the results obtained from different calculations of the same thing [64]. Outcomes in correctly conducted functional test experiments are partially attained in research by following the scientific evidence strategy, rendering QUAN analysis dispersion and validity characterisation a factor that allows the report’s outcomes to give rigour to the research. Rigour refers to the extent to which researchers strive to enhance the consistency of their studies [65]. Heale and Twycross [65] identify three characteristics of reliability: homogeneity or internal consistency, steadiness and commonality. Of the Cronbach’s alpha, split-half, Guttman, Parallel and Strict parallel approaches, Cronbach’s alpha has been recognised by several researchers as the instrument of preference for basic, coefficient-based reliability assessments provided by IBM SPSS [58,65,66].

(a) Cronbach’s alpha

To determine how effectively a group of variables or items accurately captures a singular, simplistic, latent concept, Cronbach’s alpha (α) is used. Many experts propose an alpha coefficient of between 0.65 and 0.8 as a good range, whereas an alpha coefficient of less than 0.5 is considered poor—especially for ordinal measurements [66]. There is a decent level of confidence for coefficients of 0.7 and higher, and alpha values are often interpreted as follows: high = 0.90; medium = 0.70–0.90 and poor = 0.55–0.69 [65]. According to Louangrath [67], using Cronbach’s alpha to calibrate experiments is inaccurate. This is particularly amplified in non-parametric datasets, as shown in Table 5. The idea is that the instrument’s dependability must not rely on reactions after design and testing. This paper considered alternative reliability methods to Cronbach’s alpha for QUAN datasets, as shown in Table 5.

(b) Determination of the QUAN data reliability tool

Louangrath [67] proposed a set of interconnected tests for determining reliability in non-parametric data, including raw reliability estimates, Monte Carlo simulation and N.K. Landscape optimisation simulation. It immediately follows that a test of normality for this study was conducted to establish, first, if the dataset was normally distributed; then, secondly, the application of the correct tool for reliability. Others mention generalisation, such as Razali and Wah [69] and Heale and Twycross [65]. This research used a technique described by Ezie [68] for non-parametric data analysis.

### 3.10. Interview Data Processing Approach

**Figure 2:**
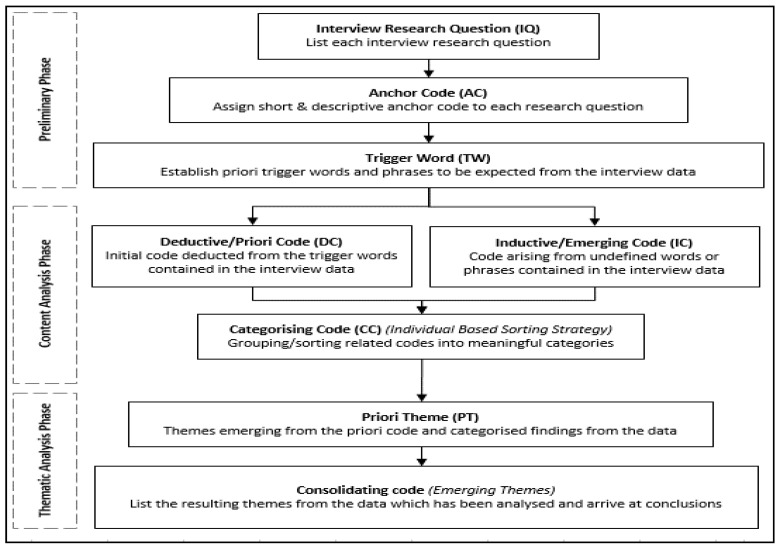
Data coding strategy (Adapted from Adu, [49]).

To extract relevant assumptions, QUAN analysis involves the statistical data analysis of many sample examples, while QUAL analysis relies on chosen semi-representative cases or descriptive representations in metanalyses [23]. The data analysis of the QUAL data was based on the inferential qualitative content analysis described by Mayring [53] and followed the coding procedure described by Adu [49] in this paper. Compiling interview transcripts is a normal first step in qualitative content research, according to Erlingsson and Brysiewicz [70] and Adu [49]. The qualitative content analysis aims to organise and summarise large amounts of material [2]. Extracting data from transcoded interviews to generate ideas or trends involves deep harvesting of data from apparent and semantic content to tacit inferences [7]. This research applied Atlas.ti^®^ technology to reduce and code data, then handle the resultant data using SPSS and M.S. Excel to display it in tables and figures for descriptive statistics. Figure 2 demonstrates the adoption process for the QUAL data coding.

### 3.11. Survey Data Processing Approach

The QUAN data statistical analysis tool chosen was SPSS. The raw data was assessed for parametric or non-parametric fit before picking a particular tool for model fit and hypothesis testing [44,53]. The analysis approach generally followed Saunders et al. [47]. Following normality tests, which were comprised principally of the degree of Skewness and Multivariate Kurtosis as guiding descriptors, correlation and regression of the model variables were applied.

(a) Model fit criterion

A generalised, structured component analysis model, such as the non-parametric model, may be used to meet model fit requirements, according to Cho et al. [71]. While model fit refers to how well a model fits the data, rather than how well the model’s variables correlate, reliability relates to how well a model matches the data. Since each model fit must statistically fulfil specific criteria before being labelled a data fit, the criterion must be defined before data collection [71].

(b) Further analysis

After the model fit criterion is met, further statistical analysis deals with the model variables and targets how the model variables behave when correlated to each other. Hypothesis testing is the last step in the model analysis, and it is performed as a critical step to test if the defined and refined study hypothesis still holds or should be rejected.

## 4. Results

In this paper, the use of the exploratory sequential approach mixed method is demonstrated in the three chosen stages (Stages 1, 2 and 3) and illustrated in Figure 1 and Table 1—which were the first stage (QUAL), where non-probability, purposive sampling was used to secure semi-structured interviews which were used to establish theories based on the conceptual model and hypothesis, followed by the two QUAN stages (2 and 3), where the survey data was collected first to establish the validity of the survey tool by conducting a pilot survey with 10% of the target sample, and then secondly to gather perspectives on top leadership commitment to H&S by conducting a multi-level perspective survey on two top leadership levels.

### 4.1. The Overall Data Collected

The QUAL study comprised *n* = 9 interviews representative of respondents in top leadership in all nine CIDB grades. In both the QUAL and the QUAN study, there was sufficient representation in the upper echelon—spanning all nine CIDB grades—and overall, several years of experience, a generally good higher education and experience in public infrastructure projects were demonstrated. Table 6 shows the participation demographic results for both the QUAL and the QUAN portions of this study.

### 4.2. Validity of the QUAL Results

The fullness of the interview questions established the preliminary interview validity and whether the intended group was attained [44]. The subsequent validity originated from the quality of the information provided—in this example, the techniques with which the answers were given, their overall depth and relevancy, and the vocabulary utilised throughout the conversation. The second crucial feature of this validation step was capturing data from the discussion to accomplish accurate transcribing that would meet the relevance criteria for the obtained data. For this research, the QUAL data collecting procedure described in the preceding sections met the criterion for this level of validity. The successive phases of validity are discussed in the sections to come.

### 4.3. Coding of the Collected Data

From the QUAL study, 387 (43 × 9) responses were collected and transcribed verbatim, then coded into 86 codes (74 deductive and 12 inductive) and 23 anchor codes. From the QUAN study, 7826 (43 × 182) responses were collected and transcribed, and then 43 codes (ordinal, 5-point Likert scale) were developed for the data for analysis.

For the QUAL study, a CAQDAS platform—Atlas.ti^®^—was used, where 414 quotations were identified from the 387 responses, resulting in a total of 86 codes developed via the content analysis of these responses. The process for categorising these codes involved reference to the questions, where a code-synthesis and categorisation process was applied consistently with the process described by Adu [49], as shown in Figure 2. The QUAN data was numerical, and the coding was performed in the survey questionnaire itself, making further coding post-data collection unnecessary.

### 4.4. Results from the Reliability Tests

Intercoder reliability in the QUAL study was achieved from 86 valid cases, where 79 agreed between the codes; disagreements comprised seven cases. A total of 172 decisions were taken. Four methods were applied simultaneously; the results achieved are presented in Table 7.

These generic results are above 0.73 on average, with the Percentage Agreement exceeding 90%, signifying that the results are all within the acceptability criteria set for each of the reliability methods defined under Section 3.8 of this paper. This provides confidence that the coding process adopted offers sufficient accuracy and relevance and that the data analysis method will render accurate results.

Similarly, reliability, validity and hypothesis testing for the QUAN study also employed a robust process, where the distribution normality test was applied, followed by correlation and regression. The distribution normality test results were achieved for validity and reliability: Kolmogorov–Smirnov Sig Index = 0.000 (non-parametric). This meant that the dataset was non-parametric. Therefore, Spearman Rank Correlation, Ordinal Logistic Regression and Multinomial generalised linear modelling were adopted and applied to the dataset for statistical analysis.

### 4.5. Results from the Statistical Analysis

For this study, a statistical analysis of the QUAL dataset was not conducted because it followed the content analysis method; nonetheless, the statistical analysis of the collected QUAN dataset was robust and yielded the following summarised results:

- Spearman rank correlation results: Rho LC to CF = 0.421; ST = 0.101; LC = 1.000; CC = 0.239; NC = 0.317; CO = −0.184
- Ordinal logistic regression: Pseudo R-square (Nagelkerke) index = 0.593; Deviance Sig = 1.000; Chi-square Sig = 0.000

The model fit data from both the correlation and regression tests demonstrated that the model fit the data well and that there was a positive correlation between the independent variable, the factor and all the covariates—except for the H&S culture outcomes variable, which was seen to be a residual variable from the outcomes of the top leadership H&S commitment.

### 4.6. Hypothesis Testing

Multinomial generalised linear modelling was applied for hypothesis testing on the QUAN dataset, focusing on the model construct. The following results were achieved: Wald Chi-square Sig LC-CF = 0.023; S.T. = 0.261; CC = 0.000; CO = 0.427. This signifies that the conditions for rejecting the null hypothesis associated with the top leadership style were not met, and the null hypothesis was therefore not rejected. All the other hypotheses were not rejected. Simply put, the practical methods provided by styles and models in developing the critical elements required in top leadership did not add value to organisational H&S outcomes.

### 4.7. Descriptive Statistics

The descriptive statistics results from both the QUAL and QUAN studies are summarised and themed as focus areas of theory as follows: H&S as a core organisational leadership function; top leadership type and style impact; top leadership H&S commitment; top leadership contextual H&S competence; the effect of national and industry contextual setting and the H&S culture outcomes. Since there are many of these tables, this paper does not intend to discuss such results; however, it aims to demonstrate the processes to be followed.

## 5. Findings

The findings from the data collected from the QUAL study resulted in the refinement and revision of the initial hypothesis. This revised hypothesis was then tested using the model construct and data collected in the QUAN study, resulting in one of the hypotheses being rejected while the remaining were not dismissed. This signifies the importance of employing a robust tool consisting of a series of consistency tests to ensure that the presence of errors in research is minimised. The top leadership effect on the H&S function has been pivotal, hence the overwhelming number of valid responses and participation in a study that questions their interest in H&S and overall involvement in the field.

The other finding is demonstrated in the choice of robust tools and how they were applied differently in both the QUAL and the QUAN study. The normality test was significant in ensuring that the assumptions of simply applying Cronbach’s alpha to any dataset, as an example, were omitted. This is a particularly useful point of departure in dataset analysis, particularly in non-parametric datasets.

## 6. Discussion

### 6.1. Convergence of the Applied Research Tool 

Firstly, the choice of the exploratory sequential approach in mixed-method research that focused on the contextual top leadership interventions in construction H&S became very useful during the reliability stages of the QUAN data, where the test of normality results revealed that the dataset was non-parametric before the selection of the appropriate reliability tool. This reinforces the assertion by Cresswell [3] and Edmonds and Kennedy [1] of the benefits offered by this type of approach.

Secondly, the model complexity of the resultant model characteristics—because the contextual setting of top leadership is uncertain of the H&S culture’s consequences—required that the coding method adopted offer very good accuracy, and this has been demonstrated in the intercoder reliability of the QUAL data, which adopted a robust, multi-tool process that demonstrated very good outcomes. This reinforces the assertion by Cresswell [3], Bairagi and Munot [5] and Palm III [4], who emphasise accuracy in the validation process.

Thirdly, an all-rounded process, as described by Zikmund et al. [19], where multiple tools are applied to ensure valid results, was set up first by the QUAL study, which sought to refine the applicability of the theory on the conceptual model and hypothesis by interviewing the subject-matter experts (in this regard, top leaders)—a process which then limited the number of respondents to ensure saturation (*n* = 9) was met, following Guest et al. [31] and Galvin [32]. This was then followed by the QUAN study, divided into two sections which were to establish the validity of the survey tool by conducting a pilot survey with 10% of the target sample and to gather perspectives on top leadership commitment to H&S by conducting a multi-level perspective survey of two top leadership levels.

### 6.2. The Impact of the Tool on Research

A practical approach that may be used for the external validity of this model is an analytical generalisation. Analytic generalisation is when case studies are applied to a theory. Then the outcomes of those case studies are acknowledged as a basic guideline for that concept, strengthening the hypothesis confirmability and its practical significance [72]. Typically, in verification procedures, assumptions are utilised for evaluating models and methods [73]. For validity to be carried out effectively in case studies, the collected data serves as evidence, and this evidence should be collected from at least five sections of the model—namely: the internal structure of the model; the variable connectedness to each other; the process of the responses; the content of the test and the implications of the test [74].

In his paper, the model structure is already described; thus, the case study would need to demonstrate the outcomes from the standpoint of the contractor and top leader(s) being evaluated. It is critical to establish a benchmark before modifying the participants; thus, the study outcomes have demonstrated the current status of the top leader in H&S functioning and their importance in H&S matters.

### 6.3. Future Study Focus

This paper established a mixed-method approach that can be applied to contextual top leadership interventions in construction H&S by adopting an exploratory sequential approach. The method itself was the paper’s focus. The in-depth details of certain aspects such as the statistical analysis, descriptive analysis, the data coding process, and the theory of top leadership in the construction H&S were not discussed but highlighted. The description of the tool is sufficient for its adoption by other researchers in the future. Future studies are encouraged, and scholars are highly invited to familiarise themselves with the methodological tool established in this study and utilise it in comparable studies and general practice to advance research and knowledge.

### 6.4. Contribution Made by This Study

A rigorous approach for designing an exploratory, sequential research method using both interviews and survey data was created in this work. The tool’s novelty was established in its point of departure from the norm in applying reliability tools prior to testing for normality and applying a rigorous process of multi-tool intercoder reliability, which also adopted a web-based tool to augment the spreadsheet calculations. Using generalised linear modelling in a study of this kind also signifies a point of departure from the norm.

The main highlights of this tool are that it is effectively managed to handle non-parametric, QUAN/QUAL data by offering a robust coding approach, data validation, model fit and reliability approaches that can be applied consistently in similar QUAN/QUAL data. The tool further offered validation capabilities for QUAN data and multi-variate hypothesis testing in an exploratory sequential method for dealing with data for H&S research of this kind; this study on the establishment of contextual top leadership interventions in construction H&S was made successful by applying this approach.

## 7. Conclusions

This paper provides guidelines for using the exploratory sequential approach in mixed-method research to effectively deal with contextual issues based on non-parametric modelling data in top leadership H&S interventions. The main focus was established by extensively providing elements that demonstrated rigour in a qualitative and quantitative study in a mixed-method environment and by distinctly placing a sequential study into the relevant context for this type of research.

The key contribution of this paper is the provision of a novel process marked by the intricacies of the reliability approaches adopted and the type of modelling analysis incorporated into the study. More specifically, this contribution can be summarised thus:

1. In the QUAL phase, the intercoder analysis was marked by a multi-tool approach augmented by a web-based platform. This demonstrated a robust method for approaching the reliability of such data in which the harmonious agreement of various tools provides a higher level of trust in the chosen approach.
2. In the QUAN analysis phase, the application of the test of distribution was appropriately placed to enable the selection of the reliability tool early in the analysis process, ensuring correctness in selecting the reliability test tool.
3. A significant point of departure from a multitude of methods in the analysis of QUAN data was the qualification of the use of Cronbach’s alpha on the dataset after the distribution test to ensure that its merits for testing such datasets were established and justified.
4. The QUAL data coding approach summarised in Figure 2 is novel and anchored on established approaches arising from extensive literature on coding.
5. The consistent application of different tools to the model, comprised of a non-parametric dataset, provided a significant advantage in applying such tools in datasets that are similar to this one in research. This further validated the propositions by Ezie [68] on the approaches to be adopted in such research.

The impact provided by the methodological tool presented in this paper is therefore established to be novel and offers a distinct advantage in the body of knowledge.

## Figures and Tables

**Table 1:** Functional content measures for the typology.

*Functional Capacity Measure (Variables)*	Functional Rules of Engagement
*Leadership type/style*	Leadership type/style influences contextual H&S competence training
*Contextual H&S competence*	Contextual H&S competence training alters Top leadership commitment
*Top leadership commitment*	Contextual H&S competence varies with Top leadership commitment
*National and industry context/setting*	National and industry context/setting influences Top leadership commitment
*Main (Transformation) element*	Critical competency elements resulting from Top leadership commitment alters the Organisational Culture and H&S Culture
*Virtuous circle (reinforcement element)*	Contextual H&S competence training varies with the H&S outcomes resulting from the H&S Culture

Source: Gogo and Musonda [26].

**Table 2:** Techniques for probing which can be used during interviews.

Probing Technique	Description of the Technique
Baiting	The researcher indicates that they are informed of specific facts, encouraging the respondent to elaborate more.
Echo	The researcher reinforces the respondent’s argument and helps them effectively enhance it.
Leading	The researcher raises a query, asking the respondent to justify their logic.
Long question	The researcher requests a fairly lengthy query, which implies that they seek a comprehensive explanation.
Silent	The researcher stays still, encouraging the respondent to speak their thoughts aloud.
‘Tell me more.’	The researcher specifically requests the respondent, despite using repetition, to elaborate on a specific topic or question.
Verbal agreement	The researcher shows curiosity in the viewpoints of the respondent through words like ‘uhhuh’ or ‘yeah, all right.’

Source: Adopted from Whiting [36].

**Table 4:** Intercoder reliability coefficient acceptability.

Stage	Description	Specifics for This Study
1	<0.00	Poor agreement
2	0.00–0.20	Slight agreement
3	0.21–0.40	Fair agreement
4	0.41–0.60	Moderate agreement
5	0.61–0.80	Substantial agreement
6	0.81–1.00	Almost perfect agreement

Source: Landis and Koch [61].

**Table 5:** Type of data distribution.

	Data Distribution	Normally Distributed Likert-Scale Data	Not-Normally Distributed Likert-Scale Data
1	Method of analysis	Parametric method	Non-parametric method
2	Reliability tool	Cronbach’s alpha	Generalization
3	Stability tool	Linear regression	Ordinal logistic regression
4	Validity tool	Pearson correlation	Spearman rank correlation

Source: Adopted from Ezie [68].

**Table 6:** Demographic.

No	Demographic Item	Interview Study	Survey Study
1	Contractor CIDB Grade	1 × 9 Grades	CIDB grade 9 = 23; 8 = 24; 7 = 20; 6 = 18; 5 = 23; 4 = 17; 3 = 22; 2 = 18; 1 = 17
2	Position in company	3 × CEO; 5 × Executive Director; 1 × Site manager	20 × CEO; 24 × Executive Director; 34 × Site Director; 42 × Site manager; 29 × Project/GM; 33 × Asst Construction Manager
3	Experience	3 × over 10 years; 3 × 6-10 years; 2 × 2–5 years; 1 × less than 1 year.	84 × over 10 years; 73 × 6–10 years; 22 × 2–5 years; 3 × less than 1 year.
4	Education	4 × Diploma; 2 × Postgrad Degree; 2 × Bachelors; 1 × Other (Cert)	63 × Diploma; 61 × Postgrad Degree; 50 × Bachelors; 7 × Other (Cert); 1 × Matric
5	Discipline of education and types of projects	3 × Engineering; 3 × Construction; 2 × Other (H.R./Commerce); 1 × Science	103 × Public Infrastructure dev.; 37 × Property dev.; 22 × Private property dev.; 16 × Mining Infrastructure dev.; 4 × Other

**Table 7:** Demographic.

No	Percentage Agreement	Scott’s Pi	Cohen’s Kappa	Krippendorff’s Alpha (Nominal)	*n* Agreements	*n* Disagreements
Variable 1 (cols 1 and 2)	91.9%	0.725	0.725	0.726	79	7
Variable 2 (cols 3 and 4)	91.9%	0.738	0.738	0.739	79	7
Variable 3 (cols 5 and 6)	91.9%	0.738	0.738	0.739	79	7
Variable 4 (cols 7 and 8)	91.9%	0.738	0.738	0.739	79	7
Variable 5 (cols 9 and 10)	91.9%	0.725	0.725	0.726	79	7
Variable 6 (cols 11 and 12)	91.9%	0.725	0.725	0.726	79	7
Variable 7 (cols 13 and 14)	90.7%	0.707	0.707	0.708	78	8
Variable 8 (cols 15 and 16)	90.7%	0.707	0.707	0.708	78	8
Variable 9 (cols 17 and 18)	91.9%	0.725	0.725	0.726	79	7
Average	**92%**	**0.73**	**0.73**	**0.73**	**79**	**7**

## Data Availability

The data supporting the reported results can be received upon reasonable request, in accordance with the data policy of the University of Johannesburg and the prevailing legislation on data sharing.

## References

[1] Edmonds W.A., Kennedy T.D., Edmonds W.A., Kennedy T.D. (2017). Explanatory-Sequential Approach. An Applied Guide to Research Designs: Quantitative, Qualitative, and Mixed Methods.

[2] Cooper D.R., Schindler P.S. (2013). Business Research Methods.

[3] Cresswell J.W. (2017). Research Design: Qualitative, Quantitative and Mixed Methods Approaches.

[4] Palm W.J. (2020). System Dynamics.

[5] Bairagi V., Munot M.V. (2019). Research Methodology: A Practical Scientific Approach.

[6] Heesen R., Bright L.K., Zucker A. (2019). Vindicating methodological triangulation. Synthese.

[7] Flick U. (2018). An Introduction to Qualitative Research.

[8] Breitbart M.M., Clifford N., French S., Valentine G. (2010). Participatory research methods. Key Methods in Geography.

[9] Bajpai N. (2016). Business Research Methods.

[10] Symonds J.E., Gorard S. (2010). Death of mixed methods? Or the rebirth of research as a craft. Eval. Res. Educ..

[11] Neuman W.L. (2014). Social Research Methods.

[12] Crotty M. (1998). The Foundations of Social Research—Meaning and Perspective in the Research Process.

[13] Maliwatu E. (2018). Understanding Context with Related Risks and Opportunities—A South African Construction Industry Perspective.

[14] Clarke J.R. (2005). Research Models and Methodologies.

[15] Askarzai W., Unhelkar B. (2017). Research Methodologies: An Extensive Overview. Int. J. Sci. Res. Methodol..

[16] Dumrak J., Mostafa S., Kamardeen I., Rameezdeen R. (2013). Factors associated with the severity of construction accidents: The Case of South Australia. Australas. J. Constr. Econ. Build..

[17] Marle F., Vidal L. (2016). Managing Complex, High-Risk Projects: A Guide to Basic and Advanced Project Management.

[18] Pepper S.C. (1942). World Hypothesis: A Study in Evidence.

[19] Zikmund W.G., Babin B.J., Carr J.C., Griffin M. (2013). Business Research Methods.

[20] Ichikawa J.J. (2017). The Routledge Handbook of Epistemic Contextualism.

[21] Morris E. (1988). Contextualism: The world view of behavior analysis. J. Exp. Child Psychol..

[22] Kothari C.R., Gaurav G. (2015). Research Methodology: Methods & Techniques.

[23] Deb D., Dey R., Balas V.E. (2019). Engineering Research Methodology: A Practical Insight for Researchers.

[24] Lavrakas P.L., Traugott M.W., Kennedy C., Holbrook A.L., de Leeuw E.D., West B.T. (2019). Experimental Methods in Survey Research: Techniques That Combine Random Sampling with Random Assignment.

[25] Elangovan N., Rajendran R. (2015). Conceptual Model: A Framework for Institutionalising the Vigor in Business Research.

[26] Gogo S., Musonda I. Leading safety culture from the top: A typology for top leadership safety commitment. Proceedings of the Joint CIB W099 & W123 Annual International Conference 2021: Good Health, Changes & Innovations for Improved Wellbeing in Construction.

[27] Babbie E. (2015). The Practice of Social Research.

[28] Patten M.L., Newhart M. (2018). Understanding Research Methods: An Overview of the Essentials.

[29] Barnsbee L., Barnett A.G., Halton K., Nghiem S., Gregory S.D., Stevens M.C., Fraser J.F. (2018). Cost-effectiveness. Mechanical Circulatory and Respiratory Support.

[30] Brisebois M., Johnstone C. (2016). System and Method for Managing and Identifying Subject Matter Experts. U.S. Patent.

[31] Guest G., Bunce A., Johnson L. (2006). How many interviews are enough?: An experiment with data saturation and variability. Field Methods.

[32] Galvin R. (2015). How many interviews are enough? Do qualitative interviews in building energy consumption research produce reliable knowledge?. J. Build. Eng..

[33] Rouse M.J., Daellenbach U.S. (2010). Rethinking research methods for the resource-based perspective: Isolating sources of sustainable competitive advantage. Strategy Manag. J..

[34] Bischoping K. (2009). Review of Social Research Methods: Qualitative and Quantitative Approaches.

[35] Arksey H., Knight P.T. (1999). Interviewing for Social Scientists: An Introductory Resource with Examples.

[36] Whiting L.S. (2008). Semi-structured interviews: Guidance for novice researchers. Nurs. Stand..

[37] Scheele B., Groeben N. (1988). Dialog-Konsens-Methoden zur Rekonstruktion Subjektiver Theorien [Dialogue Consensus Methods for the Reconstruction of Subjective Theories].

[38] Graneheim U.H., Lundman B. (2004). Qualitative content analysis in nursing research: Concepts, procedures and measures to achieve trustworthiness. Nurse Educ. Today.

[39] Kim H., Sefcik J.S., Bradway C. (2017). Characteristics of Qualitative Descriptive Studies: A Systematic Review. Res. Nurs. Health.

[40] Van De Walle D. (1997). Development and validation of a work domain goal orientation instrument. Educ. Psychol. Meas..

[41] Hayes A.F., Krippendorff K. (2007). Answering the call for a standard reliability measure for coding data. Commun. Methods Meas..

[42] Hay I., Clifford N., French S., Valentine G. (2010). Ethical practice in geographical research. Key Methods in Geography.

[43] Miller D.C., Salkind N.J. (2002). Handbook of Research Design and Social Measurement.

[44] Krippendorff K. (2018). Content Analysis: An Introduction to Its Methodology.

[45] Strauss A., Corbin J. (1998). Basics of Qualitative Research: Techniques and Procedures for Developing Grounded Theory.

[46] Sarker S., Lau F., Sahay S. (2001). Using an adapted Grounded Theory approach for inductive theory building about virtual team development. Data Base Adv. Inf. Syst..

[47] Saunders M., Lewis P., Thornhill A. (2019). Research Methods for Business Students.

[48] Hayashi P.J., Abib G., Hoppen N. (2019). Validity in Qualitative Research: A Processual Approach. Qual. Rep..

[49] Adu P. (2019). A Step-by-Step Guide to Qualitative Data Coding.

[50] Gale N.K., Heath G., Cameron E., Rashid S., Redwood S. (2013). Using the framework method for analysing qualitative data in multi-disciplinary health research. BMC Med. Res. Methodol..

[51] Freelon D.G. (2010). ReCal: Intercoder Reliability Calculation as a Web Service. Int. J. Internet Sci..

[52] Neuendorf K.A. (2012). The Content Analysis Guidebook.

[53] Mayring P. (2014). Qualitative Content Analysis: Theoretical Foundation, Basic Procedures and Software Solution.

[54] Everitt B.S., Skrondal A. (2010). The Cambridge Dictionary of Statistics.

[55] Krippendorff K. (2011). Computing Krippendorff’s Alpha-Reliability.

[56] Bird T.J., Bates A.E., Lefcheck J.S., Hill N.A., Thomson R.J., Edgar G.J., Stuart-Smith R.D., Wotherspoon S., Krkosek M., Stuart-Smith J.F. (2014). Statistical solutions for error and bias in global citizen science datasets. Biol. Conserv..

[57] McHugh M.L. (2012). Interrater reliability: The kappa statistic. Biochem. Med..

[58] Graham M., Milanowski A., Miller J. (2012). Measuring and Promoting Inter-Rater Agreement of Teacher and Principal Performance Ratings.

[59] Schreier M. (2012). Qualitative Content Analysis in Practice.

[60] Wang W. (2011). A Content Analysis of Reliability in Advertising Content Analysis Studies.

[61] Landis J.R., Koch G.G. (1977). The Measurement of Observer Agreement for Categorical Data. Biometrics.

[62] Sim J., Wright C.C. (2005). The Kappa Statistic in Reliability Studies: Use, Interpretation, and Sample Size Requirements. Phys. Ther..

[63] Mukherjee S.P., Sinha B.K., Chattopadhyay A.K. (2018). Statistical Methods in Social Science Research.

[64] Hoyt W.T., Melby J.N. (1999). Dependability of measurement in counselling psychology: An introduction to generalizability theory. Couns. Psychol..

[65] Heale R., Twycross A. (2015). Validity and reliability in quantitative studies. Evid. Based Nurs..

[66] Goforth C. (2015). Using and Interpreting Cronbach’s Alpha.

[67] Louangrath P. (2018). Reliability and Validity of Survey Scales. Int. J. Res. Methodol. Soc. Sci..

[68] Ezie O. (2018). How to Analyse and Interpret LIKERT-SCALE Questionnaire Using SPSS.

[69] Razali N.M., Wah Y.B. (2011). Power comparisons of Shapiro-Wilk, Kolmogorov-Smirnov, Lilliefors and Anderson-Darling tests. J. Stat. Model. Anal..

[70] Erlingsson C., Brysiewicz P. (2017). A hands-on guide to doing content analysis. Afr. J. Emerg. Med..

[71] Cho G., Hwang H., Sarstedt M., Ringle C. (2020). Cutoff criteria for overall model fit indexes in generalised structured component analysis. J. Mark. Anal..

[72] Yin R.K., Mills A.J., Eurepos G., Wiebe E. (2009). Analytic Generalization. Encyclopedia of Case Study Research.

[73] Popham W.J. (2008). All About Assessment/A Misunderstood Grail. Educ. Leadersh..

[74] Yin R.K. (2018). Case Study Research and Applications.

